# Subclinical Left Ventricular Dysfunction Detected by Speckle-Tracking Echocardiography in Breast Cancer Patients Treated With Radiation Therapy: A Six-Month Follow-Up Analysis (MEDIRAD EARLY‐HEART study)

**DOI:** 10.3389/fonc.2022.883679

**Published:** 2022-06-28

**Authors:** Médéa Locquet, Daan Spoor, Anne Crijns, Pim van der Harst, Arantxa Eraso, Ferran Guedea, Manuela Fiuza, Susana Constantino Rosa Santos, Stephanie Combs, Kai Borm, Elie Mousseaux, Umit Gencer, Guy Frija, Elisabeth Cardis, Hans Langendijk, Sophie Jacob

**Affiliations:** ^1^ Laboratory of Epidemiology, Institute for Radiation Protection and Nuclear Safety (IRSN), Fontenay-Aux-Roses, France; ^2^ Department of Radiation Oncology, University Medical Center Groningen (UMCG), University of Groningen, Groningen, Netherlands; ^3^ Department of Radiation Oncology, Institut Catala Oncologia (ICO), Girona, Spain; ^4^ Centro Cardiovascular da Universidade de Lisboa (CCUL), Faculdade de Medicina da Universidade de Lisboa, Lisbon, Portugal; ^5^ Department of Radiation Oncology, Technical University of Munich (TUM-MED), Munich, Germany; ^6^ Department of Radiology, Paris-Descartes University and INSERM970, Hôpital Européen Georges Pompidou, Paris, France; ^7^ Barcelona Institute of Global Health (ISGlobal), Barcelona, Spain; ^8^ Pompeu Fabra University, Barcelona, Spain; ^9^ Spanish Consortium for Research and Public Health (CIBERESP), Instituto de Salud Carlos III, Madrid, Spain

**Keywords:** MEDIRAD, breast cancer, radiotherapy, cardiac dysfunction, dosimetry, strain imaging, EARLY-HEART cohort

## Abstract

**Background:**

In the case of breast cancer (BC), radiotherapy (RT) helps reduce locoregional recurrence and BC-related deaths but can lead to cardiotoxicity, resulting in an increased risk of long-term major cardiovascular events. It is therefore of primary importance to early detect subclinical left ventricular (LV) dysfunction in BC patients after RT and to determine the dose–response relationships between cardiac doses and these events.

**Methods:**

Within the frame of the MEDIRAD European project (2017–2022), the prospective multicenter EARLY‐HEART study (ClinicalTrials.gov Identifier: NCT03297346) included chemotherapy naïve BC women aged 40–75 years and treated with lumpectomy and adjuvant RT. Myocardial strain analysis was provided using speckle‐tracking echocardiography performed at baseline and 6 months following RT. A global longitudinal strain (GLS) reduction >15% between baseline and follow-up was defined as a GLS-based subclinical LV dysfunction. Individual patient dose distributions were obtained using multi-atlas-based auto-segmentation of the heart. Dose-volume parameters were studied for the whole heart (WH) and left ventricle (LV).

**Results:**

The sample included 186 BC women (57.5 ± 7.9 years, 64% left-sided BC). GLS-based subclinical LV dysfunction was observed in 22 patients (14.4%). These patients had significantly higher cardiac exposure regarding WH and LV doses compared to patients without LV dysfunction (for mean WH dose: 2.66 ± 1.75 Gy versus 1.64 ± 0.96 Gy, *p* = 0.01). A significantly increased risk of subclinical LV dysfunction was observed with the increase in the dose received to the WH [ORs from 1.13 (V_5_) to 1.74 (D_mean_); *p <*0.01] and to the LV [ORs from 1.10 (V_5_) to 1.46 (D_mean_); *p <*0.01]. Based on ROC analysis, the LV-V_5_ parameter may be the best predictor of the short-term onset of subclinical LV dysfunction.

**Conclusion:**

These results highlighted that all cardiac doses were strongly associated with the occurrence of subclinical LV dysfunction arising 6 months after BC RT. Whether measurements of GLS at baseline and 6 months after RT combined with cardiac doses can early predict efficiently subclinical events occurring 24 months after RT remains to be investigated.

## Introduction

Breast cancer (BC) among women represents a public health challenge due to its rising incidence and its life-threatening consequences (1). Prescribed to reduce local recurrence and BC‐related mortality, radiation therapy (RT) has widely demonstrated effectiveness in the treatment of BC (2). However, radiation-induced adverse effects in healthy tissues could occur. Cardiotoxicity resulting from incidental irradiation of the heart in BC patients is now better documented (3). Indeed, BC RT leads to an increased risk of long-term major adverse cardiovascular events (MACEs), mainly coronary heart diseases, as well as excess cardiovascular (CV) mortality rates (3, 4). Up to several decades, the relative risk of clinically significant cardiac events ranged from 1.2 to 3.6 after RT (5). Darby et al. (2013) showed an incidence of acute coronary events increased by 7.4% per Gray (Gy) of mean heart dose already within 5 years following RT, later confirmed by van den Bogaard et al. (2017) who found an incidence of 16.5% per Gy (6, 7) in the first 9 years. Other authors suggested a 0.04 (95% CI: 0.02–0.06) excess relative risk per Gy received at the whole heart (8). However, the asymptomatic phase between acute heart damage occurring early after RT and the longer-term onset of MACEs leads to an underrecognized CV risk during the clinical management of BC patients immediately following RT (9).

Therefore, early screening for subclinical CV changes following RT could prove beneficial for asymptomatic patients who could nevertheless have subclinical left ventricle (LV) dysfunction. According to the American Society of Echocardiography and the European Association of Cardiovascular Imaging, oncological cardiotoxicity is diagnosed when the left ventricular ejection fraction (LVEF) is reduced by ≥10% points to below 53% after RT (10). However, the myocardial deformation [i.e., global longitudinal strain (GLS), measured by two-dimensional (2D) speckle-tracking echocardiography] appeared to be an earlier marker of subclinical LV dysfunction. Specifically, strain imaging characterizes cardiac wall deformation considering speckles. Therefore, a reduction in LVEF reflects late and advanced myocardial injury in relation to substantial cardiac damage (11). Therefore, the measurement of GLS appears to be more sensitive and relevant for detecting early LV dysfunction before the onset of LVEF deterioration, and in identifying a population at greater risk of longer-term CV morbidity and mortality (12, 13).

The ability of GLS to detect cardiotoxicity early has been little investigated among BC patients treated with RT. Some studies have shown that a statistically significant reduction of the GLS can be detected in BC women from a few weeks to 12 months following RT (14–17). However, it remains to be determined whether the observed reduction can be considered clinically relevant. Negishi et al. suggested that a reduction of GLS >15% compared with baseline appears to be clinically meaningful to highlight post‐RT cardiotoxicity, but this GLS cutoff limit was scarcely applied in onco-cardiology research (18). Moreover, few studies investigated the dose‐dependent relationship between RT and changes in GLS. In 2019, Walker et al. investigated the clinical relevance of the reduction of GLS in 79 BC patients included in the BACCARAT study (14) by defining a subclinical LV dysfunction as a relative reduction of GLS >10%. A dose–response relationship was observed, and the risk of subclinical LV dysfunction was increased by 37% per 1 Gy of mean heart dose. Nevertheless, the association was no longer statistically significant after adjustment for age, body mass index (BMI), hypertension, hypercholesterolemia, and endocrine therapy, and the study suffered from its small size and statistical power (19).

Therefore, within the frame of the European MEDIRAD project, the multi-center EARLY-HEART cohort study was designed to investigate early cardiac changes arising after BC RT in the largest population ever studied, using three approaches based on echocardiography, cardiac MRI and heart CT, and computed tomography coronary angiography (20). The present manuscript originally focuses on the specific purpose of evaluating the impact of RT (using individual patient dosimetry) on subclinical LV function changes (using speckle-tracking echocardiography) occurring in the first 6 months after BC RT. This study will open many research possibilities to find markers of early subclinical LV dysfunction potentially predicting long-term MACEs.

## Materials and Methods

### Reporting

The guidelines proposed by the Strengthening the Reporting of Observational Studies in Epidemiology (STROBE) statement were applied to the manuscript (21).

### The EARLY-HEART Study Design

As part of the MEDIRAD project (http://www.medirad-project.eu/), the multi-center EARLY-HEART study was launched in 2017. This observational study consisted of the prospective follow-up of a cohort of BC patients treated with RT over two time points post-RT (i.e., 6 and 24 months). The detailed protocol has already been described elsewhere and registered at ClinicalTrials.gov (identifier NCT03297346) (20).

The main goal of the EARLY-HEART study was to explore the relevance of several cardiac biomarkers to early identify radiation-induced subclinical dysfunction in women with unilateral left- or right-sided BC. For this purpose, both imaging biomarkers (i.e., echocardiography, computed tomography coronary angiography, and magnetic resonance) and blood-circulating biomarkers were assessed at baseline and at 6 months following RT. The current article focuses on the assessment of subclinical dysfunction post‐RT using 2D speckle-tracking echocardiography at the 6-month follow-up.

Patients were included from 5 European investigation centers: the Clinique Pasteur (Toulouse, France) for the Institut de Radioprotection et de Sûreté Nucléaire (IRSN; Fontenay-aux-Roses, France), the Universitair Medisch Centrum Groningen UMCG; Groningen, Netherlands), the Klinikum Rechts der Isar der Technischen Universität München (TUM-MED; Munich, Germany), the Institut Català d’Oncologia (ICO; Girona, Spain), and the Centro Cardiovascular da Universidade de Lisboa (CCUL; Lisbon, Portugal).

### Breast Cancer Women Population

All women aged 40–75 years with histologically diagnosed unilateral left- or right-sided stage I–III invasive adenocarcinoma of the breast or ductal carcinoma *in situ* (DCIS) and treated with adjuvant RT after breast-conserving surgery in one of the 5 investigating centers could be included. In addition, women had to be chemotherapy naïve. Non-inclusion criteria were previous thoracic or mediastinal radiation, previous CV diseases, and current pregnancy and/or lactation. Abnormal cardiac imaging exams after inclusion were considered as dismissal criteria. Specifically for echocardiography, an LVEF <50%, suggesting an alteration of the cardiac function before RT, was set as a dismissal criterion.

### Radiation Therapy Treatment

All patients underwent adjuvant radiotherapy following the lumpectomy. According to the center, three-dimensional conformal radiotherapy (3D-CRT), volumetric modulated arc therapy (VMAT), and/or fixed-field intensity-modulated radiotherapy (IMRT) was performed.

Different fractionation schedules were used according to patient and center specificities: mainly 25 fractions/50 Gy following a standard protocol or 15 fractions/40.5 Gy following a hypofractionated protocol. A boost dose was delivered to the tumor site in some patients (with a maximum of 14.49 Gy administered). Deep inspiring breath-holding was recommended in some patients with a heart close to the anterior chest wall and in all left-sided patients followed at the UMCG center. The patient treatment was normalized and optimized according to the statement of the International Commission on Radiation Units and Measurements (ICRU) and in compliance with QUANTEC dose constraints (5).

### Individual Patient Dosimetry

Cardiac structure delineation was performed centrally by the UMCG using multi-atlas based automatic segmentation of the heart and its substructures previously published by Spoor et al. (22). This technique reduces inter-observer variability during the delineation of cardiac volumes. Two contoured cardiac structures were considered in our analysis: the whole heart (WH) and the left ventricle (LV), their relevance being highlighted in previous research. The exact planned radiation dose was reconstructed from the delineated volumes and three-dimensional dose-volume parameters were obtained for each patient. In the current analysis, mean dose (D_mean_, in Gy), minimum dose (D_min_, in Gy), and maximum dose (D_max_, in Gy) were studied as well as relative volumes of the WH and LV receiving at least 5 Gy (V_5_, in %) and 20 Gy (V_20_, in %), both suggested as good prognostic parameters of cardiac complications (14, 23).

### Cardiac Examinations

Two-dimensional speckle-tracking trans-thoracic echocardiography, a recent semi-automated imaging technique, was performed before RT and at the 6-month follow-up. The level of deformation between systole and diastole is expressed in percentage and will be negative in the presence of shortening (24). Subsequently, longitudinal shortening will engender negative values. A weakened myocardium is described by a reduced systolic function followed by a smaller decline between systole and diastole. The strain value is then reduced and closer to zero (24). Left lateral decubitus position was required for the exam performed by a trained and qualified cardiologist or technician. Different measurement techniques were used between the different institutes (Siemens, Philips, or General Electric). Different software was used to calculate strain values. LVEF was determined using Simpson’s biplane method during three sets of measurements (mean was reported) (25). Other conventional measurements have been collected: left ventricular end-diastolic volume, left ventricular end-systolic volume, E/A wave ratio, tricuspid annular plane systolic excursion, tricuspid annular S wave, left ventricular outflow tract diameter, left ventricular outflow tract velocity time integral, heart rate, and cardiac output. By tracking movements of myocardial speckles occurring during 3 cardiac cycles including an apical 4-, 3-, and 2-chamber view, the 2D speckle-tracking echocardiography also provided systolic strain values (26). GLS (%) and GLS rate (s^−1^) have been recorded. A >15% relative percentage reduction from the initial GLS value was considered a clinically relevant marker of subclinical LV dysfunction as suggested in 2016 by the European Society of Cardiology (10). Based on LVEF, subclinical LV dysfunction was defined according to Cancer Therapy-Related Cardiac Dysfunction (CTRCD) definition for patients with a reduction in LVEF ≥10% from baseline to a final value less than 53% after RT (27). Images with poor echogenicity were excluded as well as patients without echocardiography imaging available at the two time points. All ultrasound data were collected at each center by operators blinded to all other clinical data, including radiotherapy treatment modalities.

### Non-Radiation CV Risk Factors

In addition to BC treatment characteristics, information on clinical patients’ characteristics were collected at baseline, particularly the CV risk factors such as age, BMI, smoking status, hypertension, diabetes, cholesterol, menopausal status, and statin consumption.

### Statistical Analysis

All quantitative variables were expressed as mean (µ) ± standard deviation (SD). Group comparisons were carried out using a *t*-test in case of normal distribution (checked using the Shapiro–Wilk test) or a nonparametric Wilcoxon–Mann–Whitney test in case of skewed distribution. Qualitative variables were reported in absolute (*n*) and relative (%) frequencies and were compared using *χ*² or Fisher’s exact tests. Paired Wilcoxon signed-rank tests were applied to assess changes in echocardiography parameters before RT and 6 months post-RT. The impact of baseline characteristics (i.e., age, smoking status, hypertension, obesity, diabetes, total cholesterol level, and hormonotherapy) on the risk of subclinical LV dysfunction was explored using a binary logistic regression yielding odds ratio (OR) and the 95% confidence interval (95% CI). The relationship between dose-volume parameters and subclinical LV dysfunction was also investigated using univariate (crude model) and multivariate (adjusted model) binary logistic regressions. An adjustment was made for age, smoking status, hypertension, total cholesterol level, and hormonal therapy. Obesity and diabetes were not included in the model to avoid the strong correlation with total cholesterol levels. To determine which dose-volume parameter best discriminates between BC patients at risk of subclinical LV dysfunction and those not at risk, areas under the curve were obtained using receiver operating characteristic analysis (AUROC). An AUROC between 0.5 (no discriminative power) and 1 (perfect discriminative power) is essential for clinical testing (28). The AUROC values of the different dose-volume parameters were statistically compared using the method of Delong et al. (29). Optimal cutoffs were calculated according to Youden’s index. The 5% critical threshold was set to consider statistically significant results. In regression models, because of multiple testing, the significance level was further corrected in 0.05/k (Bonferroni correction). All analyses were performed using R version 4.0.3 software.

## Results

### Description of the Studied Population

**Figure 1:**
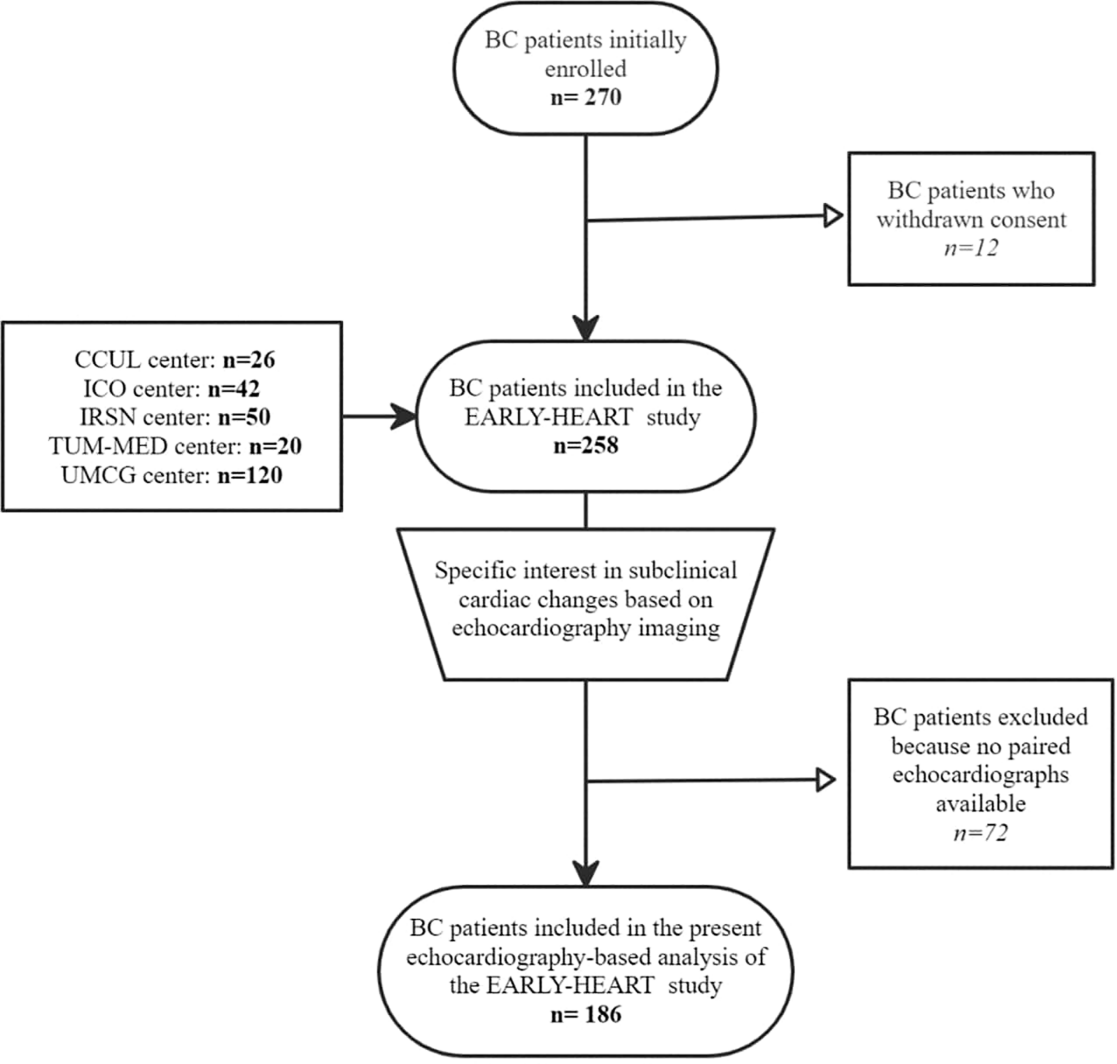
Flowchart of BC patient inclusion and exclusion in the EARLY-HEART study based on echocardiography.

The 5 European centers included a total of 258 BC patients. For the present study based on echocardiography parameters, 186 BC patients were analyzed, 72 being excluded due to the absence of paired echocardiography data available (i.e., before RT and 6 months post-RT). A detailed flowchart is available in **Figure 1**.

The 186 BC women had a mean age of 57.5 ± 7.9 years. Baseline characteristics of the 186 BC patients are described in **Table 1**. Left-sided BC represented 64% of the sample, 14.5% were obese, a few BC women were affected by diabetes mellitus (4.3%), and more than half were non-smokers (52.7%). A large sample suffered from an invasive (78.0%) grade 2 (51.9%) carcinoma. Patients were mainly treated by 3D-CRT (60.2%), with a 15 fraction/40.05 Gy (33.9%) protocol; 35.5% received a boost, and 65% received hormonal therapy.

**Table 1:** Baseline characteristics of the 186 BC women from the EARLY‐HEART cohort included in the echocardiography-based analysis.

Characteristics	µ ± SD or *n* (%)
Clinical and cardiovascular data	
Age, years	57.5 ± 7.9
Body mass index, kg/m²	25.5 ± 4.1
Menopausal status, yes	137 (74.9)
Onset of menopause, years	11.4 ± 7.4
Cardiovascular treatment, yes	23 (12.4)
Statins prescription, yes	20 (10.7)
Obesity, yes	27 (14.5)
Total cholesterol, mg/dl	209.7 ± 47.2
Triglycerides, mg/dl	106 ± 56.1
Diabetes mellitus, yes	8 (4.3)
Hypertension, yes	41 (22.0)
Smoking status	
No	98 (52.7)
Former	58 (31.2)
Current	30 (16.1)
Former or current smoker, pack-year	14.3 ± 12.3
Breast cancer information	
Laterality, left	119 (64.0)
Invasive breast carcinoma, yes	145 (78.0)
Breast carcinoma *in situ*, yes	101 (54.3)
Grade of breast cancer	
Grade 1	66 (36.1)
Grade 2	95 (51.9)
Grade 3	22 (12.0)
Radiotherapy protocol	
Type of radiotherapy	
3D-CRT	112 (60.2)
IMRT	2 (1.1)
VMAT	72 (38.7)
Fraction/total RT dose	
15/40.05 Gy	63 (33.9)
16/42.56 Gy	36 (19.3)
20/47 Gy	25 (13.4)
2 5/50 Gy	39 (21.0)
Lymph node radiation, yes	11 (5.9)
Breath-hold RT, yes	66 (35.5)
Boost, yes	95 (51.1)
Total boost dose, Gy	11.9 ± 1.9
Other breast cancer treatment	
Hormonotherapy	
No	65 (35.1)
Tamoxifen Aromatase inhibitors	72 (38.9) 48 (25.9)

BC, breast cancer; SD, standard deviation.

### Cardiac Radiation Dosimetry

The cardiac dose-volume parameters are reported in **Table 2**. D_mean_ to WH and D_mean_ to LV dose were 1.76 ± 1.16 Gy and 2.09 ± 1.91 Gy, respectively, with higher dose-volume parameters for left-sided BC than for right-sided BC (*p*-value < 0.001).

**Table 2:** Dose-volume parameters for the whole heart and left ventricle.

	Whole BC patients (*n* = 186)		Left-sided BC patients (*n* = 119)		Right-sided BC patients (*n* = 67)		
Dosimetry	µ ± SD	Range	µ ± SD	Range	µ ± SD	Range	*p*-value
**Whole heart**							
D_mean_ (Gy)	1.76 ± 1.12	0.14–6.76	2.21 ± 1.17	0.14–6.76	0.97 ± 0.34	0.28–2.02	<0.0001
D_min_ (Gy)	0.33 ± 0.25	0.00–1.20	0.38 ± 0.28	0.00–1.20	0.25 ± 0.16	0.00–0.80	<0.0001
D_max_ (Gy)	23.6 ± 18.5	0.88–55.4	33.9 ± 15.0	0.88–55.4	5.41 ± 5.07	2.16–29.9	<0.0001
V_5_ (%)	3.80 ± 5.66	0.00–31.3	5.84 ± 6.21	0.00–31.3	0.21 ± 0.72	0.00–5.20	<0.0001
V_20_ (%)	1.03 ± 1.95	0.00–12.2	1.63 ± 2.25	0.00–12.2	0.01 ± 0.06	0.00–0.50	<0.0001
**Left ventricle**							
D_mean_ (Gy)	2.09 ± 1.91	0.04–8.18	2.97 ± 1.87	0.07–8.18	0.53 ± 0.30	0.04–1.60	<0.0001
D_min_ (Gy)	0.50 ± 0.31	0.00–1.61	0.64 ± 0.28	0.00–1.61	0.26 ± 0.17	0.00–0.83	0.005
D_max_ (Gy)	18.2 ± 18.4	0.23–55.2	27.9 ± 16.5	0.25–55.2	1.13 ± 0.70	0.23–5.35	<0.0001
V_5_ (%)	5.34 ± 8.12	0.00–36.8	8.38 ± 8.84	0.00–36.8	0.00 ± 0.00	0.00–0.00	<0.0001
V_20_ (%)	1.49 ± 3.16	0.00–14.2	2.35 ± 3.71	0.00–14.2	0.00 ± 0.00	0.00–0.00	<0.0001

BC, breast cancer; SD, standard deviation; Gy: Gray.

### Echocardiography Parameters

Conventional echocardiography parameters at baseline and 6 months post-RT are shown in **Table 3**. No significant changes were shown between those parameters before and after RT (all *p*-values >0.05). LVEF-based LV dysfunction defined by a ≥10% reduction in LVEF from baseline to <53% after RT was found in 6 patients (3.2% of the sample).

**Table 3:** Description of conventional echocardiography parameters before RT and RT+6 months.

Echocardiography parameters	Before RT	RT+6 months	*p*-value
Left ventricular ejection fraction, %	62.3 ± 6.1	61.5 ± 6.6	0.08
Left ventricular end-diastolic volume, ml	77.4 ± 18.8	76.9 ± 19.2	0.90
Left ventricular end-systolic volume, ml	30.1 ± 10.2	30.1 ± 9.6	0.67
E/A wave ratio	1.05 ± 0.52	1.03 ± 0.31	0.97
Tricuspid annular plane systolic excursion, cm	3.21 ± 4.10	2.39 ± 0.33	0.15
Tricuspid annular S wave, cm/s	13.29 ± 2.49	13.47 ± 2.52	0.32
Left ventricular outflow tract diameter, mm	20.13 ± 3.87	19.93 ± 2.26	0.36
Left ventricular outflow tract velocity time integral, cm	22.5 ± 4.79	22.55 ± 4.10	0.56
Heart rate, beats per minute	68.1 ± 9.04	68.6 ± 11.4	0.82
Cardiac output, L/min	4.79 ± 2.50	4.29 ± 1.62	0.17

RT, radiation therapy.

Regarding the strain imaging, by considering GLS and GLS rate as continuous variables, no significant changes were observed between baseline and 6 months post-RT (all *p*-values >0.05) (**Table 4**). Subclinical LV dysfunction, defined as a relative reduction of GLS >15%, was observed in 11.8% of the total sample (i.e., 22 patients). Among the 22 women with subclinical LV dysfunction, 4 had a right‐sided BC (18.2%) and 18 had a left-sided BC (81.8%) (*p*-value = 0.21). Among the 6 patients with LVEF-based LV dysfunction, 5 patients (83.3%) had a reduction of GLS >15%.

**Table 4:** Global longitudinal strain and strain rate parameters before RT and at RT+6 months.

	GLS (%)	GLS rate (s^−1^)
Before RT	−19.4 ± 3.2	−1.08 ± 0.20
RT+6 months	−19.2 ± 3.6	−1.09 ± 0.34
*p*-value	0.82	0.13

RT, radiation therapy.

### Relationships Between a Reduction of GLS >15% and Clinical or Radiation Parameters

The impact of baseline characteristics on the risk of a reduction of GLS >15% at the 6-month follow-up was analyzed (**Table 5**). Higher total cholesterol levels increased the risk of subclinical LV dysfunction (OR = 1.02 [1.01–1.03]). However, no other usual CV risk factors were associated (all *p*-values >0.05) with GLS reduction. Parameters of the RT protocol also affected the onset of a subclinical LV dysfunction: the RT protocol (i.e., fraction × total dose) increased the risk by 4.32-fold (95% CI of the OR: 1.33–16.8), irradiation of lymph nodes by 5.55-fold (95% CI of the OR: 1.27–23.0), and a boost by 2.83-fold (95% CI of the OR: 1.09–8.32) (all *p‐*values <0.05).

**Table 5:** Univariate logistic regressions exploring the relationship between baseline characteristics and a relative reduction of GLS >15% occurring 6 months after BC RT.

Characteristics	OR (95% CI)	*p*-value
**Clinical and cardiovascular data**		
Age, years	1.00 (0.94–1.06)	0.99
Menopause, yes	0.84 (0.32–2.57)	0.77
Cardiovascular treatment, yes	0.29 (0.02–1.56)	0.99
Obesity, yes	1.05 (0.23–3.51)	0.94
Total cholesterol, mg/dl	1.02 (1.01–1.03)	0.02
Triglycerides, mg/dl	1.00 (0.99–1.01)	0.66
Diabetes mellitus, yes	4.23 (0.53–27.1)	0.12
Hypertension, yes	0.89 (0.24–2.63)	0.84
Smoking status, yes	1.01 (0.90–1.08)	0.97
**Breast cancer information**		
Laterality, left	2.30 (0.80–8.34)	0.31
Hormonotherapy No (reference) Tamoxifen Aromatase inhibitors	1.23 (0.47–3.63) 1 0.64 (0.17–2.28) 2.15 (0.73–6.79)	0.69 0.49 0.17
**Radiotherapy protocol**		
3D-CRT, yes VMAT, yes	2.15 (0.74–7.78) 0.47 (0.13–1.37)	0.19 0.20
Fraction/total RT dose 15/40.05 Gy (reference) 16/42.56 Gy 20/47 Gy 25/50 Gy	1 1.10 (0.20–5.33) 4.40 (0.90–21.7) 4.32 (1.33–16.8)	0.90 0.06 0.02
Lymph node radiation, yes	5.55 (1.27–23.0)	0.02
Breath-hold RT, yes	0.33 (0.09–0.95)	0.04
Boost, yes	2.83 (1.09–8.32)	0.04

RT, radiation therapy; GLS, global longitudinal strain; BC, breast cancer; Gy, Gray; 3D-CRT, three-dimensional conformal radiotherapy; VMAT, volumetric modulated arc therapy.

**Figure 2:**
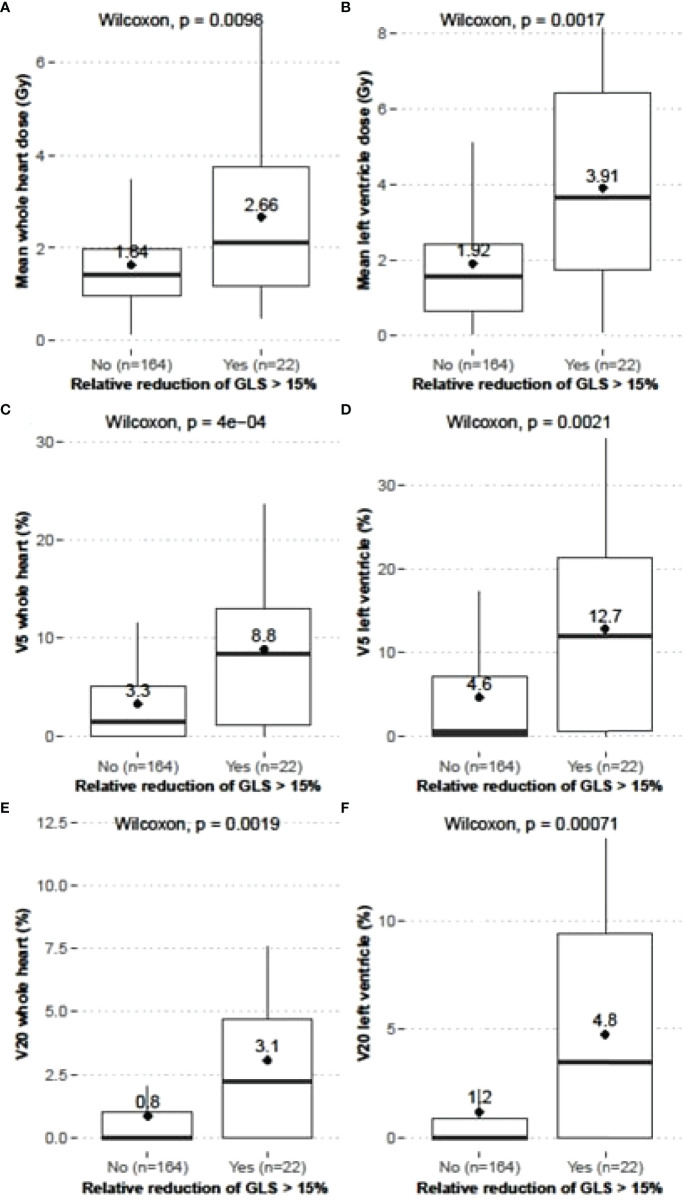
Comparisons of dose-volume parameters between BC patients with or without a reduction of GLS >15%; D_mean_ to WH **(A)** and LV **(B)** (Gy), V_5_ of WH **(C)** and LV **(D)**, and V_20_ of WH **(E)** and LV **(F)**; mean values: numerical and ♦.

Comparisons between dose-volume parameters obtained for the WH and the LV were performed between patients with or without relative reduction of GLS >15%. A significantly higher mean dose was observed in patients with a relative reduction of GLS >15% (**Figure 2**).

The same observation was made regarding V_5_ (%) and V_20_ (%) (**Figure 2**). Further analysis was undertaken to determine the magnitude of the association between dose-volume parameters and the reduction of GLS >15% (**Table 6**).

**Table 6:** Relationship between dose-volume parameters and a relative reduction of GLS >15% occurring 6 months after BC RT highlighted by binary logistic regressions and ROC analyses (*n* = 186 patients from the EARLY-HEART cohort).

Dosimetry	Crude OR (95% CI)	*p*-value	Adjusted^a^ OR (95% CI)	*p*-value	Adjusted^a^ AUROC (95% CI)	*p*-value
**Whole heart**						
D_mean_ (Gy)	1.85 (1.31–2.68)	0.0005	1.74 (1.20–2.61)	0.005	0.794 (0.688–0.902)	0.02
D_min_ (Gy)	0.24 (0.02–1.86)	0.20	0.36 (0.02–3.49)	0.40	0.575 (0.492–0.655)	0.28
D_max_ (Gy)	1.04 (1.01–1.07)	0.0009	1.03 (0.99–1.06)	0.02^b^	0.765 (0.651–0.878)	0.004
V_5_ (%)	1.14 (1.06–1.23)	0.0004	1.13 (1.05–1.23)	0.002	0.813 (0.712–0.914)	0.001
V_20_ (%)	1.47 (1.21–1.81)	0.0001	1.39 (1.13–1.75)	0.003	0.804 (0.703–0.906)	0.001
**Left ventricle**						
D_mean_ (Gy)	1.51 (1.23–1.88)	0.00008	1.46 (1.17–1.87)	0.003	0.807 (0.704–0.909)	0.004
D_min_ (Gy)	3.59 (0.80–16.33)	0.09	5.11 (0.91–3.30)	0.07	0.629 (0.457–0.706)	0.06
D_max_ (Gy)	1.04 (1.01–1.06)	0.005	1.03 (1.01–1.06)	0.02^b^	0.770 (0.660–0.878)	0.003
V_5_ (%)	1.10 (1.05–1.16)	0.0001	1.10 (1.05–1.17)	0.001	0.815 (0.717–0.914)	0.005
V_20_ (%)	1.26 (1.12–1.42)	0.00009	1.20 (1.07–1.37)	0.003	0.808 (0.701–0.909)	0.004

^a^Model adjusted for age, smoking status, hypertension, total cholesterol level, and hormonotherapy.

^b^No longer after Bonferroni correction for multiple tests (significant threshold: α/k).

All dose-volume parameters to WH and LV (D_mean_, V_5,_ and V_20_) were significantly associated with a reduction of GLS >15% (all *p*-values <0.001), except for D_min_ and D_max_ (after adjustment). For both cardiac structures, the associations remained significant after adjustment for covariates and multiple statistical testing (all *p*-values <0.05). In the adjusted model, each increase of 1 Gy of D_mean_ to WH increased the risk of a reduction of GLS >15% by 74% and each increase of 1 Gy of D_mean_ to LV increased the risk by 46%. Moreover, each additional percent of V_5_ and V_20_ increased the risk of subclinical LV dysfunction by 13% and 39% (WH) and by 10% and 20% (LV), respectively. Sensitivity analysis had been undertaken using a reduction of GLS >10% to define a subclinical LV dysfunction (**Supplementary Table 1**). A reduction of GLS >10% was highlighted in 36 patients (i.e., 23.5% of the total sample). Similar conclusions were drawn using this cutoff. **Supplementary Table 2** also highlights the consistency of our results when applying a one-way sensitivity analysis omitting one center at a time.

**Table 6** provides information about the ability of dose-volume parameters to early identify BC patients at risk of subclinical LV dysfunction occurring 6 months following RT. All parameters showed an AUROC value higher than the point with no discriminant power (i.e., 0.500) except for D_min_. The highest AUROC value was observed for V_5_ regardless of the cardiac structure (i.e., 0.813 for WH and 0.815 for LV). Also, AUROC values of V_20_ (i.e., 0.804 for WH and 0.808 for LV) showed a very satisfactory discriminative power. However, the AUROC values of all the dose-volume parameters did not differ between them (all *p*‐values >0.05). Therefore, no dose-volume parameter statistically performed better than another. In addition to the dose–response relationship showing the relevance of heart dose for GLS reduction with a risk gradually increasing with higher doses, we evaluated the optimal cutoff of dose parameters to predict the risk of subclinical LV dysfunction-based ROC analysis. D_mean_ >2.74 Gy to the WH was the mean dose from which the classification of our patients between the two groups (i.e., with or without subclinical LV dysfunction) was the most accurate. Regarding LV, a D_mean_ >3.1 Gy was established. For V_5_, the threshold was set at >5.2% (WH) and >8.4% (LV).

## Discussion

Designed to early identify cardiotoxicity in BC women treated with RT, the EARLY-HEART study suggested a strong relationship between cardiac absorbed dose and the occurrence of subclinical LV dysfunction at 6 months following RT based on >15% reduction in GLS estimated by echocardiography.

The mean value of GLS in the whole population did not significantly decrease from baseline to 6 months post-RT. Other authors previously showed significant GLS changes after BC RT, with a mean reduction of the GLS following RT of 5% in Erven et al., 6% in Walker et al., and 7.9% in Trivedi et al. (follow-up from 3 to 12 months) (14, 16, 17). In these studies, significant changes were highlighted in left-sided BC only.

GLS damage was further studied as a relative change in each individual and from a clinical perspective (10). A binary clinical endpoint of subclinical LV dysfunction was set by categorizing BC patients with or without reduction of GLS >15% as previously suggested in order to be largely beyond the possible errors related to the accuracy and reproducibility of measurements (18). Among the 186 women, 22 presented a subclinical LV dysfunction (11.8%). The prevalence of subclinical LV dysfunction was slightly higher when applying the cutoff of 10% (19.3%). Although not negligible, these two rates were lower than those obtained in other studies (applying the 10% cutoff) where they ranged from 27.5% to 46.8% (14, 16, 17). The high proportion of right-sided BC, cardiac dose differences, and the chemotherapy-naïve status of BC women in our study may explain this lower rate. Fourati et al., using similar study criteria, also obtained a lower prevalence rate of cardiac dysfunction (6.8%) (30) (i.e., 1.76 ± 1.12 Gy versus in our study versus 2.8 Gy of mean dose in the study of Fourati et al.; 22% of left-sided BC versus 42% of right-sided BC).

The EARLY-HEART study robustly showed a relationship between dose-volume parameters and an increased risk of subclinical LV dysfunction (adjusted ORs ranging from 1.13 [1.05–1.23] (V_5_) to 1.74 [1.20–2.61] (D_mean_) for the WH structure and from 1.10 [1.05–1.17] (V_5_) to 1.46 [1.17–1.87] (D_mean_) for the LV structure). The magnitude of the association was consistent with previous studies (or even stronger): OR = 1.37 [1.01–1.86] in Walker et al. and OR = 1.04 [1.01–1.06] in Fourati et al. (both analyzing relationship between D_mean_ and a reduction in GLS >10%) (30, 31). Furthermore, three parameters were able to properly distinguish BC women at risk or not of a reduction of GLS>15%, 6 months after RT: D_mean_, D_max_, and V_5._ The lowest AUROC was 0.765 for D_max_ (WH) and the highest AUROC was 0.815 for V_5_ (LV). Then, V_5_, a dose-volume parameter, seemed highly relevant, as previously shown by other studies (7, 14). Indeed, van den Bogaard et al. showed that V_5_ (LV) was the best predictor of acute coronary events (HR = 1.016 [1.002–1.030], *p*-value = 0.016). However, the mean heart dose remains currently the most widely used predictor of cardiotoxicity (31). Furthermore, intrinsic to our sample, the threshold of 3 Gy for D_mean_, previously identified by Erven et al. (17), was also highlighted. Indeed, a D_mean_ of 2.74 Gy (WH) or a V_5_ >5.2% (WH) should not be exceeded to prevent the CV risk. The threshold of 3 Gy for D_mean_, previously identified by Erven et al. (17), was also highlighted. Some impactful studies (e.g., Darby et al., showing a dose–response relationship between acute coronary events and mean heart dose) incited RT protocols to evolve to limit the risk of MACE and cardiac doses (e.g., breath‐hold, VMAT, and hypofractionation can reduce cardiotoxicity) (6). Proton therapy may also be applied for patients still at increased risk. However, our study combining different techniques of RT showed that some patients remained in dose ranges that should be considered with caution (e.g., D_mean_ of 3 Gy to LV). Vigilance must be brought to this specific point, especially in randomized controlled trials where a systemic assessment of radiation‐induced cardiotoxicity should be investigated as a clinical endpoint.

Our study was the first to demonstrate, with sufficient statistical power, a dose-dependent relationship between early cardiotoxicity defined using the stringent and recommended criterion of a reduction of GLS >15% and a wide range of doses absorbed (inclusion of both right- and left-sided BC patients). Its prospective design allowed us to include only BC women without baseline overt CV diseases and chemotherapy and to control CV risk factors, making the results on the observation of an early subclinical LV dysfunction induced by RT more robust.

However, our study had some limitations. The interpretation of the present results must be made with knowledge of these. First, our sample of BC women was limited by strict inclusion criteria. Further studies should include a larger representation of BC patients treated with RT only (e.g., risk in younger and older BC patients, risk in patients with or without previous CV diseases, and risk according to regional specificities). Likewise, the lower proportion of left-sided BC patients in our EARLY-HEART population compared to other studies could impact the observed change in mean GLS, which was not significant. Moreover, inter-observer (i.e., different cardiologists) and inter-operator (i.e., different vendors) imprecisions cannot be excluded to explain the absence of a statistically significant decrease in mean GLS in our study even if the good reproducibility of the strain measure using echocardiography was established (32). Indeed, inter-operator relative mean errors ranged from 5.4% to 11.0% when inter-observer relative mean errors varied from 1.9% to 11.3%. These values of errors remained lower than that observed for LVEF and other conventional echocardiography parameters (32). Our one-way sensitivity analysis omitting one center at a time reduced this potential bias and showed the robustness of our findings. Finally, although the current results were adjusted for baseline CV risk factors, it cannot be ruled out that other confounding factors could impact the observed association (e.g., parental history of CV diseases, sedentary habits, and nutritional habits).

In the future, it remains to be investigated whether the occurrence of subclinical LV dysfunction observed at RT+6 months is declining, maintaining, or improving in the longer term. The specific interest in echocardiography data imaging from the EARLY‐HEART study will be further studied to determine which specific segments of the longitudinal strain (i.e., basal, mid, or apical) could be the most affected by dose-volume parameters. Indeed, Tuohinen et al. recently showed that the dose absorbed at the level of the apical region of the anterior wall of the LV was linked to a significantly higher deterioration of the GLS than in other locations (15). Furthermore, the same research team recently showed that diastolic strain rate was an earlier predictor of dysfunction than systolic LV strain rate (23), which could be of interest knowing that diastolic function is involved in diffuse fibrosis following RT.

## Conclusion

The present analysis of BC women from the EARLY-HEART study showed that the cardiac doses absorbed during RT were strongly associated with the occurrence of a subclinical LV dysfunction at 6 months after RT. Therefore, primary and secondary CV health prevention could be beneficial at this early asymptomatic phase to reduce long-term CV complications. These findings already suggest the potential relevance of an early screening of BC patients treated with RT to eventually early implement cardioprotective actions during RT by limiting the dose absorbed by the heart as much as possible.

## Data Availability Statement

The raw data supporting the conclusions of this article will be made available by the authors, without undue reservation.

## Ethics Statement

The study protocol and related amendments received approval from the competent ethics authority of each center involved (France: Comité de Protection des Personnes Sud-Ouest IV, ID: CPP2015/66/2015-A00990-69–R1, and Agence Nationale de Sécurité des Médicaments, ID: 150873B-12; the Netherlands: Medisch Ethische Toetsingscommissie van het Universitair Medisch Centrum Groningen [METc UMCG], ID: METc 2017/379, NL62360.042.17; Germany: Ethikkomission der Technischen Universität München, ID: 235/17 S; Spain: Comitè d’Etica d’Investigatio CEAi GIRONA, ID: EARLY HEART v1.1 05/07/2017 i FIP v1.3; Portugal: Comissao de Ética do Centro Hospitalar Lisboa Norte e do Centro Académico de Medicina de Lisboa [CHLN e CAML], ID: 257/2017). The patients/participants provided their written informed consent to participate in this study.

## Author Contributions

Conceptualization: SJ, AC, HL, AE, SS, KB, GF, and EC. Methodology: SJ, HL, GF, and EC. Software: ML. Validation: ML and SJ. Formal analysis, ML and SJ. Investigation: SJ, AC, ML, DS, FG, MF, SS, SC, KB, EM, and UG. Resources: GF and EC. Data curation: ML, EM, UG, SJ, and DS. Writing—original draft preparation: ML and SJ. Editing: all authors. Supervision: EC, GF, and HL. All authors contributed to the article and approved the submitted version.

## Funding

The European Community’s Horizon 2020 Programme supported the EARLY-HEART study conducted in the frame of the MEDIRAD - Implications of Medical Low Dose Radiation Exposure - project spanning from 2017 to 2021 granted by the Euratom Research and Training Programme 2014-2014 under agreement No. 755523.

## Conflict of Interest

The authors declare that the research was conducted in the absence of any commercial or financial relationships that could be construed as a potential conflict of interest.

## Publisher’s Note

All claims expressed in this article are solely those of the authors and do not necessarily represent those of their affiliated organizations, or those of the publisher, the editors and the reviewers. Any product that may be evaluated in this article, or claim that may be made by its manufacturer, is not guaranteed or endorsed by the publisher.
