# Supplementary material for: Subclinical Left Ventricular Dysfunction Detected by Speckle-Tracking Echocardiography in Breast Cancer Patients Treated With Radiation Therapy: A Six-Month Follow-Up Analysis (MEDIRAD EARLY‐HEART study)
Source: Front Oncol. 2022 Jun 28;12:883679. doi: 10.3389/fonc.2022.883679 (PMC9275564; doi:10.3389/fonc.2022.883679)
Supplement: Supplementary file 1 [file DataSheet_1.docx]

Supplementary Material

# Supplementary Tables

| **Supplementary Table 1.** Relationships between dose-volume parameters and a reduction of **GLS >10%** occurring during the 6-month follow-up after BC RT highlighted by binary logistic regressions | | | | |
| --- | --- | --- | --- | --- |
| Dosimetry | Crude OR (95%CI) | Unadjusted *p*-value | Adjusted^a^ OR (95%CI) | Adjusted^a^ *p*-value |
| Whole heart |  |  |  |  |
| D_mean_ (Gy) | 1.98 (1.46-2.83) | 0.00006 | 1.95 (1.35-2.97) | 0.0007 |
| D_min_ (Gy) | 1.21 (0.24-5.61) | 0.81 | 2.49 (0.41-15.1) | 0.31 |
| D_max_ (Gy) | 1.03 (1.01-1.05) | 0.01^b^ | 1.02 (0.99-1.05) | 0.06 |
| V_5_ (%) | 1.18 (1.09-1.28) | 0.00004 | 1.16 (1.07-1.28) | 0.0007 |
| V_20_ (%) | 1.46 (1.21-1.79) | 0.0001 | 1.42 (1.15-1.82) | 0.003 |
| Left ventricle |  |  |  |  |
| D_mean_ (Gy) | 1.50 (1.25-1.84) | 0.00003 | 1.48 (1.20-1.88) | 0.0005 |
| D_min_ (Gy) | 5.79 (1.62-22.3) | 0.0008 | 9.09 (2.06-46.0) | 0.004 |
| D_max_ (Gy) | 1.03 (1.01-1.05) | 0.008 | 1.03 (0.99-1.04) | 0.05 |
| V_5_ (%) | 1.10 (1.05-1.15) | 0.00006 | 1.09 (1.04-1.15) | 0.0004 |
| V_20_ (%) | 1.25 (1.12-1.42) | 0.00001 | 1.22 (1.08-1.40) | 0.003 |

^a^ Model adjusted for age, smoking status, hypertension, total cholesterol level, and hormonotherapy

| **Supplementary Table 2**. One-way sensitivity analysis of the relationship between dose-volume parameters and a reduction of GLS >15% by omitting one investigation center at a time | | | | | | | | | | |
| --- | --- | --- | --- | --- | --- | --- | --- | --- | --- | --- |
|  | **Excluding CCUL center (n= 165)** | | **Excluding ICO center (n=150)** | | **Excluding IRSN center (n=142)** | | **Excluding TUM-MED center (n=172)** | | **Excluding UMCG center (n=116)** | |
| Dosimetry | Adjusted^a^ OR (95%CI) | Adjusted^a^  *p*-value | Adjusted^a^ OR (95%CI) | Adjusted^a^  *p*-value | Adjusted^a^ OR (95%CI) | Adjusted^a^  *p*-value | Adjusted^a^ OR (95%CI) | Adjusted^a^  *p*-value | Adjusted^a^ OR (95%CI) | Adjusted^a^ *p*-value |
|  |  |  |  |  |  |  |  |  |  |  |
| **Whole heart** |  |  |  |  |  |  |  |  |  |  |
| D_mean_ (Gy) | 1.67 (1.16-2.51) | 0.008 | 1.63 (1.11-2.81) | 0.02^b^ | 1.76 (1.14-2.83) | 0.01 | 1.87 (1.28-2.85) | 0.002 | 2.09 (1.36-3.44) | 0.001 |
| V_5_ (%) | 1.12 (1.04-1.22) | 0.004 | 1.11 (1.02-1.21) | 0.01 | 1.13 (1.03-1.25) | 0.01 | 1.17 (1.07-1.29) | 0.001 | 1.21 (1.09-1.37) | 0.001 |
| V_20_ (%) | 1.37 (1.12-1.73) | 0.004 | 1.33 (1.07-1.69) | 0.01 | 1.24 (1.07-1.47) | 0.007 | 1.42 (1.16-1.80) | 0.001 | 1.35 (1.15-1.18) | 0.001 |
| **Left ventricle** |  |  |  |  |  |  |  |  |  |  |
| D_mean_ (Gy) | 1.42 (1.14-1.82) | 0.003 | 1.40 (1.11-1.82) | 0.006 | 1.35 (1.04-1.79) | 0.03^b^ | 1.49 (1.19-1.90) | 0.0007 | 1.44 (1.12-1.93) | 0.008 |
| V_5_ (%) | 1.09 (1.03-1.15) | 0.002 | 1.09 (1.03-1.16) | 0.005 | 1.09 (1.02-1.18) | 0.01 | 1.11 (1.05-1.18) | 0.0004 | 1.11 (1.04-1.19) | 0.001 |
| V_20_ (%) | 1.19 (1.05-1.35) | 0.006 | 1.17 (1.03-1.34) | 0.001 | 1.15 (1.04-1.29) | 0.007 | 1.22 (1.08-1.39) | 0.002 | 1.19 (1.08-1.35) | 0.002 |

^a^ Model adjusted for age, smoking status, hypertension, total cholesterol level, and hormonotherapy

^b^ No longer significant after Bonferroni correction for multiple testing (significant threshold: α/k)
